# Prevalence of *Linguatula serrata* Nymphs in Mesenteric Lymph Nodes of Cattle and Buffaloes Slaughtered in Ahvaz Abattoir, Iran

**Published:** 2013

**Authors:** AR Alborzi, P Haddad Molayan, M Akbari

**Affiliations:** 1 Department of Pathobiology, School of Veterinary Medicine, Shahid Chamran University of Ahvaz, Iran; 2 Department of Veterinary Parasitology, School of Veterinary Medicine, Shahid Chamran University of Ahvaz, Iran

**Keywords:** *Linguatula serrata*, Mesenteric lymph nodes, Cattle, Buffaloes, Iran

## Abstract

**Background:**

*Linguatula serrata*, one of the parasitic zoonoses, inhabits the canine respiratory system (final hosts). The objective of this study was to determine the prevalence rate of *L. serrata* nymphs in mesenteric lymph nodes (MLNs) of cattle and buffaloes (intermediate hosts) that were processed in the Ahvaz, Iran abattoir.

**Methods:**

During November 2010 to March 2011, 223 animals (119 cattle and 104 buffaloes), in different sex and three age groups (<2, 2–< 3 and 3-> 3 years old) were sampled randomly at Ahvaz abattoir. Up to 35 grams of their mesenteric lymph nodes were examined separately for nymphal stages of *L. serrata* by digesting the samples with acid- pepsin method, collected the nymphs and counted under stereomicroscope.

**Results:**

Overall 37(16.6%) of 223 animals were infected with *L. serrata* nymphs in their mesenteric lymph nodes. Prevalence of the infection in cattle and buffaloes were 16.8% and 16.3% respectively. The number of collected nymphs of MLNs was ranged from 1 to 16. No significant differences were seen in the infection rates between males and females (sexes) and age groups in the cattle and buffaloes (*P* <0.05).

**Conclusion:**

*Linguatula serrata* has an active life cycle in the studied area and a zoonotic potential for transmission between animal and human. Avoiding use of raw MLNs to dogs can help reduce the infection.

## Introduction

*Linguatula serrata* (Frohlich, 1789) is a cosmopolitan species of the phylum Pentastomida. The name pentastome or “five mouths” is derived from the four anterior legs like protuberances, plus a fifth median projection that actually bears the mouth (1, 2). Name of the parasite is also derived from Latin: lingua = tongue, serratus = saw like (3). Adult *L. serrata* inhabits the nasal passages and paranasal sinuses of wild and domestic canids, which serve as definitive or final hosts. Females excrete thousands eggs per day. The eggs are infectious for plant feeders (including humans), when swallowed by a suitable herbivorous animal (intermediate host), the larvae take into different organs away from the intestine such as mesenteric lymph nodes (MLNs), liver, lung, etc., in which develop to the infective nymphal stages (4). Nymphs of the parasite in the intermediate host are detected by biopsy, exploratory laparotomy, postmortem examination, and subsequent histopathology (2). The final host becomes infected by eating the infected viscera of intermediate hosts (4). It is a zoonotic parasite causing visceral linguatulosis in humans by eating the eggs that results encapsulated larvae in inner organs and also nasopharyngeal linguatulosis or ‘Halazoun syndrome’ (by eating raw or semi cooked liver, lymph nodes, etc. (3, 5). Several cases of linguatolosis in human have been reported in Iran with clinical signs of nasopharyngeal symptoms including sneezing, coughing and nasal discharge, dyspnea, dysphagia and frontal headache after eating of barbecued liver (6–8).

Several attempts have been directed to study the prevalence rate of *L. serrata* in the dogs as definitive host in Turkey, Dincer (9); Egypt, Khalil (10); Morocco, Pandey (11); Bangladesh, Rahman (12); Iran, Rezaee (13); Yagi (14); in ruminants as intermediate hosts such as in goats and sheep in Turkey (9); Sudan, El-Badawi (15); Egypt, El-Sherry (16); Bangladesh, Rahman (17); Jordan, Sherkov (18); Spain, Valero-Lopez (19); Iran, Rezaee (13), Nourollahi Fard (20); and also in bovine aminals in Iran, Tajik (21, 22), Hami (23), Nourollahi Fard (24).

Cattle and buffaloes are major dairy animals of traditional village farming systems significantly contributing to agricultural economy in Ahvaz.

The aim of this study was to determine the prevalence of *L. serrata* nymphs in mesenteric lymph nodes of cattle and buffaloes (as two intermediate hosts) slaughtered at Ahvaz abattoir, Iran.

## Materials and Methods

Ahvaz City, the capital of Khuzestan Province in south west of Iran is located at 20 m above sea level, in south west of Iran (32°20’ N, 40°20’ E) with subtropical climate condition, a moderate winter, and hot summer, with temperatures regularly at least 45 degrees Celsius, sometimes exceeding 50 degrees Celsius. The average annual rainfall is around 230 mm.

During December 2010 to March 2011, 223 animals (119 cattle and 104 buffaloes), in three age groups (<2, 2– < 3 and 3- > 3 years old) were selected randomly at Ahvaz abattoir. Mesenteric lymph nodes were sampled directly from the slaughtered animals.

After separation of all fats surrounding mesenteric lymph nodes of each sample, they were weighed and examined for nymphal stages of *L. serrata*.

The lymph nodes examination was performed in two steps. In the first, they were sliced in 4–5 mm thick and observed carefully to find the nymphs. In the second, about 35 g of mixed sliced lymph nodes were digested in 150 ml of digestion fluid containing 6 g pepsin, 2.5 g sodium chloride and 10 ml hydrochloric acid in 600 ml distilled water, and incubated at 37 °C for 48 h. Digested materials of each sample were washed with tap water by using a fine sieve. The nymphs were collected and counted under stereomicroscope. All collected nymph were maintained in 10% formalin, a fixative solution.

### Statistical analysis

All data analyses were performed using the SPSS software (version.16).To compare relative frequency of the infection between different groups, chi-square tests were used. Differences were considered significant at *P* < 0.05.

## Results

The prevalence rate of *L. serrata* nymphs in mesenteric lymph nodes of 223 cattle and buffaloes Slaughtered at Ahvaz abattoir, Iran in different sex and age twenty out of 119 cattle (16.8%) and seventeen out of 104 buffaloes (16.3%) were found to be positive. Twelve out of 69 (17.3%) male cattle and eight out of 50 (16%) female cattle had nymphs in their mesenteric lymph nodes. The prevalence of *L. serrata* nymphs in mesenteric lymph nodes of male buffaloes 11 out of 68 (16.2%) and female buffaloes 6 out of 36 (16.7%). No significant differences were seen in the infection rates between males and females (sexes) in the cattle and buffaloes (*P* <0.05). The prevalence of *L. serrata* nymphs in mesenteric lymph nodes of cattle and buffaloes in age groups (<2, 2- < 3, 3- < 3 years old) is showing Tables 1, 2. There were also no significant differences in the infection between age groups of both the animals. The number of nymphs isolated from about 35 g MLNs of examined cattle and buffaloes ranged from 1 to16 and 1to 12 respectively.

**Table 1:** The prevalence of *Linguatula serrata* nymphs in mesenteric lymph nodes of cattle Slaughtered at Ahvaz abattoir, Iran

Animals	Gender		Total	Age (yr)		
	Female	Male		<2	2 - <3	3 - >3
Cattle examined (n)	50	69	119	38	50	31
Infected Cattle (n/%)	8/16	12/17.4	20/16.8	3/7.9	9/18	8/25.8

**Table 2:** The prevalence of *Linguatula serrata* nymphs in mesenteric lymph nodes of buffaloes Slaughtered at Ahvaz abattoir, Iran

Animals	Gender		Total	Age (yr)		
	Female	Male		<2	2 - <3	3 - >3
Buffalos (n)	36	68	104	31	43	30
Infected buffalos (n/%)	6/16.7	11/16.2	17/16.3	5/16.1	7/16.3	5/16.7

## Discussion

Prevalence study on *L. serrata* infection in final (dogs) and intermediate hosts, especially ruminant animals is important of epizoology and epidemiology aspects for control program and measurements.

Occurrence of *L. serrata* infection in stray dogs have been reported from certain parts of Iran with a prevalence of 62.2% being recorded in central part, Sahrekord (25), 76.5% in southern, Shiraz (26) and 27.83% in North West, Urmia (27). In these studies, average numbers of *L. serrata* (Mean intensity) were recorded 4.069 (with ranging from 1 to 19) and 3.81 (ranging from 1 to 13) for each infected dog from Shiraz and Urmia respectively.

The highest number of *L. serrata* per infected dog was recorded from Sahrekord with ranging from 1 to 29. *L. serrata* infection in dogs have been also reported from other country such as Lebanon (43.3% of stray dogs in Beirut), 38% in parts of India (28), 56 and 47% of male and female dogs in Sudan (14). Several different studies have been conducted in Iran to determine the prevalence of *L. serrata* infection in ruminants.

In our study from south-west of Iran, 16.8% of examined cattle (n = 119) and 16.34% of examined buffaloes (n= 104) had *L. serrata* nymphs in mesenteric lymph nodes with ranging from 1 to 16. Because of close contact between dogs and intermediate hosts (ruminants), existence of the *L. serrata* infection in these important ruminant animals (cattle and buffaloes) indicates that dogs and likely other canids in this area should be infected to the adult parasite. Therefore presence of related hosts (intermediate and final) ensures continuity of the parasite's life cycle.

Tow studies of *L. serrata* in domestic bovids from northwest of Iran (Urmia) have revealed different results. One has found the infection with a prevalence as high as 44% in native cattle (n = 110) from Slaughter- house of Urmia. (Tajik H, Tavassoli, 2006) (21).The number of isolated parasite from each infected lymph node of cattle was varied from 1 to 69 with a mean of 5.48. Other has found a prevalence rate of *L. serrata* nymphs in mesenteric lymph nodes, lung and liver of river buffaloes (n= up to 80) in Urmia as 18.75%, 2% and 2% respectively (22), the number of nymphs isolated from each MLNs of river buffaloes ranged from 1 to 6.

A prevalence of *L. serrata* infection in cattle slaughtered at Kerman slaughterhouse (southeast of Iran) has found 16.22% in mesenteric lymph nodes and 6.66% in their mediastenal lymph nodes (24).

The present and all other studies indicate that *L. serrata* is highly endemic parasite to different parts of Iran and infection rates of the parasite in ruminants can be related to types of animals, type and size of samples. Very low prevalence of the infection has been found in domestic bovids slaughtered in Tabriz abattoir, because *L. serrata* nymph infection in cattle and buffaloes were determined 0.25%, 0.5% respectively and overall 0.38% in both animals with examination of their liver and lungs (23).

Overall, infection of MLNs to *L. serrata* nymph in domestic bovids (22) and in other ruminants is usually higher than other organs, as prevalence of the infection in livers and MLNs of sheep and goats in Shiraz were reported 3.0%, 11.5% (29) and 6.4%, 29.9% (30) respectively.

However, *L. serrata* infection rate in cattle and buffaloes and their risk as intermediate hosts are lower than sheep and goats, as the infection rate of *L. serrata* nymphs in MLNs of goats in Tabriz, northwest of Iran (31) and of sheep in Urmia (32) was 27.1%,, 52.5% respectively

Based on results of the present study, the prevalence rate of *L. serrata* infection in cattle and buffaloes in southwest area of Iran seems to be like to rate of the infection in cattle from Kerman (southeast of Iran) and in buffaloes of Urmia but severity of the infection in the animals of Ahvaz very lower (with range of 1 to16 nymphs) in comparison to those in Urmia (with range of 1 to 69 nymphs).

Subtropical climate condition of Ahvaz with hot summer and decrease of rain falling in last several years seems to be main causes for decrease of eggs survival, consequently decrease of availability of them to herbivorous animals.

Buffalos are often farmed together with cattle in these regions. Considering relatively food and habitat differences between buffalos and cattle, similar infection rate in the animals may reflect similarity of exposure to parasite eggs shed into the environment through definitive hosts (especially dogs). The common cause of the infection transmission to the animals appears to be drinking water. It is necessary to control and prevention the infection with regulatory treatment of dogs, avoiding feeding raw or uncooked ruminant offal to dogs, contact with canine saliva and drinking water used by dogs.

## Conclusion

*Linguatula serrata* has an active life cycle in the studied area and a zoonotic potential for transmission between animal and human. Avoiding use of raw MLNs to dogs can help reduce the infection.
